# Immunological Biomarkers in Autism Spectrum Disorder: The Role of TNF-Alpha and Dependent Trends in Serum IL-6 and CXCL8

**DOI:** 10.3390/life14091201

**Published:** 2024-09-22

**Authors:** Catalina Mihaela Anastasescu, Veronica Gheorman, Eugen-Cristi Stoicanescu, Florica Popescu, Victor Gheorman, Ion Udriștoiu

**Affiliations:** 1 Mental Health Center for Children, Neuropsychiatry Hospital of Craiova, 200187 Craiova, Romania; catalina_tocea@yahoo.com; 2 Department of Medical Semiology, University of Medicine and Pharmacy of Craiova, 200349 Craiova, Romania; 3 Pediatry Department, Emergency Clinical Hospital Râmnicu-Vâlcea, 200300 Râmnicu-Vâlcea, Romania; prof.dr.stoicanescu@gmail.com; 4 Pharmacology Department, University of Medicine and Pharmacy of Craiova, 200349 Craiova, Romania; prof_floricapopescu@hotmail.com; 5 Department of Psychiatry, University of Medicine and Pharmacy of Craiova, 200349 Craiova, Romania; victor.gheorman@umfcv.ro (V.G.); ion.udristoiu@umfcv.ro (I.U.)

**Keywords:** autism spectrum disorder (ASD), interleukin 6 (IL-6), interleukin 8 (CXCL8), tumor necrosis factor alpha (TNF-alpha), immunological biomarkers, neuroinflammation

## Abstract

Background: Autism spectrum disorder (ASD) has seen a rise in prevalence, and the immune system’s role in brain development is increasingly recognized. This study investigates the relationship between immune dysregulation and ASD by examining serum concentrations of interleukin 6 (IL-6), interleukin 8 (CXCL8), and tumor necrosis factor alpha (TNF-alpha) in children. Methods: Serum samples from 45 children with ASD and 30 controls, aged 2 to 12 years, were analyzed using electrochemiluminescence, chemiluminescent microparticle immunoassay, and chemiluminescent immunoassay. ASD symptoms were assessed using the Autism Spectrum Rating Scale (ASRS) and Social Communication Questionnaire (SCQ). Results: No significant correlation was observed between CXCL8 levels and ASD. IL-6 levels showed a trend toward elevation in boys with ASD. TNF-alpha levels were significantly higher in children with ASD under 5 years compared to older children and controls, though no correlation with symptom severity was found. Conclusions: TNF-alpha may be a potential biomarker for early ASD detection, especially in younger children. Further research on larger cohorts is needed to understand the role of immune dysregulation in ASD.

## 1. Introduction

Cytokines play a pivotal role in modulating the intricate interplay between the immune system and the central nervous system. Emerging evidence highlights their multifaceted involvement in neural processes, including neurotransmitter function, neuroplasticity, and neuroendocrine activity [1,2,3]. Notably, pro-inflammatory cytokines have been implicated in various psychiatric conditions, such as depression, schizophrenia, bipolar disorder, and Alzheimer’s dementia [4]. Their ability to cross the blood–brain barrier and induce inflammatory responses within the brain underscores their significance in neuroimmunology [2,3,5,6,7].

Moreover, studies have shown that the immune system can influence behavioral and cognitive functions, suggesting a possible link between immunological dysregulation and psychiatric symptoms [8]. Increasing evidence points to neuroinflammation as a critical factor in the synaptopathies (disorders related to synaptic function and connectivity) observed in several psychiatric disorders. This growing body of research supports the immunological theory of psychiatric conditions such as psychoses and autism spectrum disorder (ASD), where neuroinflammatory processes have been increasingly recognized as key contributors to their pathophysiology [9,10,11].

Focusing specifically on ASD, interleukin 6 (IL-6), traditionally classified as a pro-inflammatory cytokine, has a dual role that includes regenerative and anti-inflammatory activities, leading to complex effects on metabolic and neuronal processes [12,13]. Immunocytochemical studies have revealed the activation of microglia and astroglia, along with increased production of cytokines like macrophage chemoattractant protein (MCP)-1 and transforming growth factor beta 1 (TGF-β1) in neuroglia [14].

Additionally, research has identified a correlation between abnormal behaviors in ASD and elevated levels of cytokines such as C-X-C Motif Chemokine Ligand 8 (CXCL8) and IL-12p40, further implicating neuroinflammatory processes in the pathogenesis of ASD [8,15]. Moreover, maternal immune activation (MIA) during pregnancy has emerged as a significant risk factor for neurobiological changes in offspring that may predispose them to psychiatric disorders, including ASD [16,17,18,19].

Compelling evidence from animal studies supports the translational relevance of these findings. MIA has been shown to induce neurodevelopmental deficits that resemble those observed in humans [20,21]. Mechanistically, elevated levels of pro-inflammatory cytokines such as IL-6 and tumor necrosis factor alpha (TNF-alpha) have been linked to synaptic dysregulation and ASD-like symptoms [22,23,24]. Furthermore, post-mortem analyses of ASD brains have revealed changes in neuroinflammatory markers, highlighting the importance of neuroimmunological processes in ASD pathophysiology [25].

What sets this study apart from previous research is its focus on identifying distinct immunological subtypes within the ASD population. By examining the specific plasma concentrations of IL-6, CXCL8, and TNF-alpha in children with ASD, this study seeks to uncover patterns that may differentiate subgroups of ASD based on their immune profiles. This approach not only enhances our understanding of the heterogeneous nature of ASD but also paves the way for personalized interventions tailored to the unique immunological characteristics of each subtype. The integration of clinical and biochemical analyses in this study provides a novel perspective that could lead to more targeted therapeutic strategies, distinguishing it from previous studies that have often focused on broader immune-system markers without such detailed subgroup analysis.

Given the accumulating evidence linking cytokines to ASD, this study aims to explore the immunological underpinnings of ASD by examining plasma concentrations of IL-6, CXCL8, and TNF-alpha in children with ASD compared to neurotypical counterparts. We hypothesize that children with ASD will exhibit higher plasma concentrations of IL-6, CXCL8, and TNF-alpha compared to neurotypical controls. By integrating clinical and biochemical analyses, we seek to identify immunological subtypes of ASD, which could provide insights into potential therapeutic avenues and personalized interventions.

## 2. Materials and Methods

### 2.1. Informed Consent

The study adhered to the principles outlined in the Declaration of Helsinki and the Code of University Ethics. Approval for the study protocol was obtained from the University and Scientific Ethics and Deontology Commission of the University of Medicine and Pharmacy in Craiova (Approval No. 154/24 September 2021). Informed consent was obtained from all parents or legal guardians for the children’s participation in the study and the publication of resulting data. Additionally, acceptance forms were signed by all relatives of the participating children. Biological samples were collected by a certified laboratory.

### 2.2. Statistical Analysis

Statistical analyses were performed using IBM SPSS version 23. Graphical representations were generated using MS Office Excel 2016 and Word 2016 on a Windows 10 operating system. Comparative analyses were conducted using Student’s *t*-test for groups with approximately Gaussian distributions, with a significance threshold of 0.05. For groups that did not follow Gaussian distributions, the Mann–Whitney U test was used as an alternative to Student’s *t*-test. In cases requiring multiple comparisons, ANOVA tests were employed with a significance threshold of 0.05 alongside post-hoc tests, such as the Tamhane Test, Dunnett’s test, and Tukey’s HSD test, depending on the equality of variances. For non-parametric distributions requiring multiple comparisons, the Kruskal–Wallis test was utilized. Chi-square tests were employed to assess qualitative correlations between variables. Pearson correlation was applied for comparative analyses in groups with approximately normal distributions, while Spearman correlation coefficients were used to investigate correlations between variables that did not follow normal distributions.

Age-group classification was established using cluster discriminant analysis with ANOVA and Wilk’s Lambda, resulting in two subgroups for each category: study group (autistic patients) <5 years, study group (autistic patients) >5 years, control group (healthy subjects) <5 years, and control group (healthy subjects) >5 years.

### 2.3. Study Groups

This case–control study was conducted between 2021 and 2022, involving a cohort of 75 children aged 2 to 12 years, comprising both boys and girls. Of these, 45 children belonged to the study group, while 30 children formed the control group.

To determine if there were statistical differences in sex, age, and residence between the two groups, we applied a *t*-test for unequal variances. The results are presented in Table 1. We did not find statistically significant differences in age distribution. Regarding sex distribution, our groups had a ratio of 1.77:1 for boys versus girls. In terms of geographic location, the majority of participants hailed from urban areas, with a smaller group from rural areas, all located in the southern region of Romania. The difference in terms of residence was statistically significant (*p* = 0.031).

### 2.4. Inclusion and Exclusion Criteria

Participants in the study group were recruited from children who underwent evaluation and diagnosis of autism spectrum disorder (ASD) between 2017 and 2022 at a certified private medical office in Craiova, Romania, accredited by both the Dolj Public Health Directorate and the Romanian College of Physicians, Dolj branch. The selection criteria were meticulously outlined in the study information form and included children aged 2 to 12 years diagnosed with ASD based on established diagnostic criteria, such as those outlined in the DSM-5; children with ASD who had not been administered with medication for at least two months preceding sample collection, nor had they received any medical interventions that might impact laboratory results; and children with ASD whose parents or legal guardians provided informed consent for their participation in the study.

Exclusion criteria encompassed children with significant medical conditions that could potentially influence the study outcomes; children on medications affecting the inflammatory system; those falling outside the specified age range of 2 to 12 years; individuals who were agitated and, because of agitation, were unable to provide laboratory samples; and children whose parents declined to sign the acceptance form for study inclusion or who withdrew from the study at any stage. Agitated patients were excluded solely because we were unable to collect the necessary samples. We acknowledge that this exclusion may introduce potential bias in the results, as it could exclude those with more severe ASD symptoms. This limitation is addressed in the limitations section to provide a transparent account of the study’s strengths and weaknesses.

Volunteers from the general population were recruited for the control group and had similar age, sex, and background criteria. Legal guardians were thoroughly informed about the nature and purpose of the clinical trial prior to enrolment.

### 2.5. Clinical Assessments

The Social Communication Questionnaire (SCQ) and Autism Spectrum Rating Scale (ASRS) were employed to assess the 45 children included in the study group, aiming to analyze cytokine levels and ASD symptomatology. We used the translated and adapted versions of the SCQ and ASRS, which have been validated for the Romanian population. These assessments were meticulously administered by a pediatric psychiatrist with an authorized license for the SCQ and ASRS and were completed with the assistance of the parents or legal guardians who provided responses. A significant SCQ score is defined as exceeding 15 points [26]. The ASRS encompasses a comprehensive set of evaluation scales designed to measure behaviors associated with ASD. This scale is applicable to children aged 2 to 18 years and includes the ASRS scale (comprising social/communication, unusual behaviors, and self-regulation scales), the DSM-IV-TR scale, and treatment scales (peer socialization, adult socialization, social/emotional reciprocity, atypical language, stereotypes, behavioral rigidity, sensory sensitivity, attention/self-regulation for the 2–5-year-old scale, and attention for the 6–18-year-old scale) [27].

#### Validation of ASD Diagnosis in Very Young Children

Diagnosing autism spectrum disorder (ASD) in very young children, particularly those as young as 2 years old, presents significant challenges due to the variability in early developmental behaviors and the subtle presentation of symptoms at this stage. Recognizing the importance of early and accurate diagnosis, our study employed a multi-faceted approach to ensure the reliability of ASD diagnoses within this age group.

1. Rigorous Diagnostic Criteria:

All children included in the study group were diagnosed based on the criteria outlined in the Diagnostic and Statistical Manual of Mental Disorders, Fifth Edition (DSM-5). The DSM-5 provides a comprehensive framework for diagnosing ASD, focusing on persistent deficits in social communication and interaction, as well as restricted, repetitive patterns of behavior, interests, or activities. These criteria are well-established and widely accepted in clinical practice, ensuring a standardized approach to diagnosis.

2. Multidisciplinary Evaluation:

Each diagnosis was made by a multidisciplinary team of specialists, including pediatric neurologists, psychiatrists, and psychologists with extensive experience in early childhood ASD diagnosis. This collaborative approach ensured that the diagnosis was not based on a single assessment but was corroborated by multiple experts, each contributing their specialized knowledge to the final diagnosis.

3. Consideration of Developmental Trajectories:

Although our study primarily focused on a single time point, many children were observed over an extended period as part of their ongoing clinical care. This longitudinal observation allowed clinicians to monitor the consistency of ASD symptoms over time, providing additional validation for the initial diagnosis. The stability of the diagnosis over time, even in very young children, supports the reliability of our early diagnostic assessments.

4. Parental Involvement and Reporting:

Parents or legal guardians played a crucial role in the diagnostic process, providing essential information about their child’s early development, behavior, and any concerns they had observed. The use of validated questionnaires, such as the Social Communication Questionnaire (SCQ) and the Autism Spectrum Rating Scale (ASRS), which have been adapted and validated for the Romanian population, further ensured the accuracy of the reported symptoms and behaviors.

In summary, while diagnosing ASD in children as young as 2 years old is challenging, our study employed a rigorous and validated approach to ensure the accuracy and reliability of these diagnoses. The combination of standardized diagnostic tools, a multidisciplinary team, and parental involvement provides a strong foundation for the validity of our findings, even in this very young cohort. Future studies with larger samples and longitudinal follow-ups will be essential to further confirm these early diagnoses and understand their long-term implications.

### 2.6. Collection and Analysis of Laboratory Samples

Sample collection took place between October 2021 and January 2022 at the Collection Center of the Bioclinica Laboratory in Craiova, Romania. Patients and volunteers arrived early in the morning after an overnight fast on a scheduled date.

For interleukin 6 (IL-6) analysis, 1 mL of serum was collected from each patient. The serum was subsequently frozen at −20 °C and transported in dry ice under optimal conditions to the Bioclinica Laboratory in Timișoara, Romania, where the tests were conducted. The electrochemiluminescence method (ECLIA) was utilized for analysis, which involves electron-transfer reactions generating excited states and emitting light. The reference value for serum IL-6 concentration was set at <7 pg/mL.

In the case of interleukin 8 (CXCL8), a minimum of 1 mL of serum was collected from each study participant. Due to the need for specialized analysis, the Bioclinica Laboratory froze the serum at −20 °C and transported it in dry ice under optimal conditions to an accredited medical laboratory of the German Limbach Laboratory Group, specifically Labor Dr. Limbach U. Kollegen, Heidelberg, Germany. Analysis was performed using chemiluminescent microparticle immunoassay (CMIA) with Fourier transform infrared spectroscopy (FT-IR) as the detection method. The reference value for serum CXCL8 concentration was established at <62 pg/mL.

For tumor necrosis factor alpha (TNF-alpha), 1 mL of serum was collected from each patient and processed similarly to the IL-6 samples. The Bioclinica Laboratory in Timișoara performed the tests using the immunochemical method with chemiluminescence detection (CLIA). CLIA combines chemiluminescent systems with immunoreactions to determine analyte concentrations via chemical probes that generate light. The reference value for serum TNF-alpha concentration was ≤8.1 pg/mL.

Laboratory results were obtained within 10 to 15 working days after sample collection.

## 3. Results

### 3.1. Results for Interleukin 6 (IL-6). Descriptive Analysis, and Comparisons by Age and Sex between the Study Group and the Control Group

**Figure 1:**
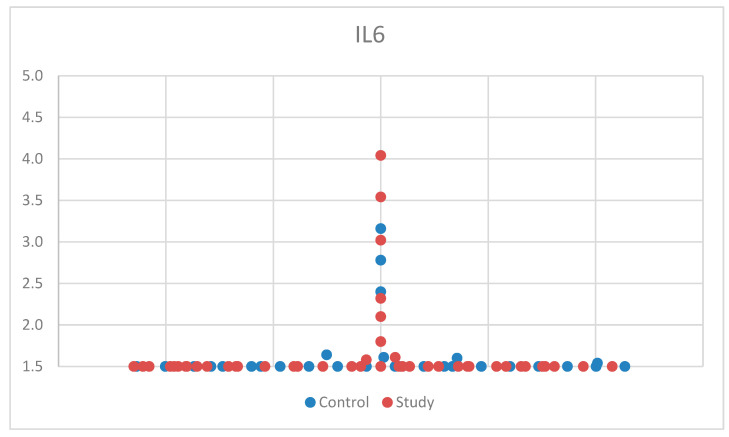
Scatter-plot of interleukin 6 (IL-6) concentrations in the study group and the control group. The majority of the values were <1.5 pg/mL, which was the lowest value that could be measured according to the laboratory testing.

For IL-6, we used the Mann–Whitney U test, a non-parametric test for non-Gaussian distribution, for comparison between the control group and the study group. One patient had aberrant results, was considered an outlier, and was excluded from the study. After applying the tests, we found no statistical significance between the two groups, *p* = 0.592 (Table S1). The median (and interquartile range) was 1.50 (1.50–1.50) (Table S4). For a better visualization of the IL-6 concentration, the obtained results are represented in Figure 1. The small sample size may have limited our ability to detect significant differences in IL-6 concentrations between groups.

#### 3.1.1. Comparisons of Serum Levels of Interleukin 6 (IL-6) by Age in the Study Group and the Control Group

For IL-6, we used the Kruskal–Wallis test for multiple comparisons due to non-parametric distribution in order to compare the serum levels of IL-6 by age (2–12 years old) between the study group and the control group.

**Figure 2:**
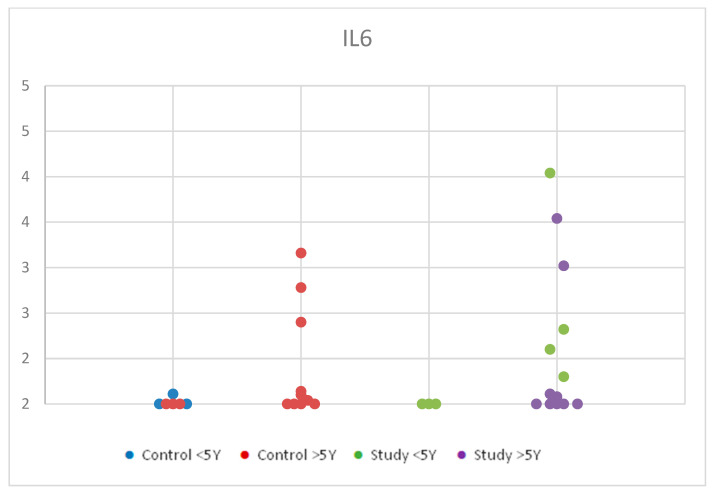
Scatter-plot of interleukin 6 (IL-6) concentrations in the study group and the control group by age between 2 and 12 years. There were four groups: Control <5 Y = control group under 5 years old, Control >5 Y = control group over 5 years old, Study <5 Y = study group under 5 years old, Study >5 Y = study group over 5 years old.

We observed a slight increase in serum concentrations from the control group under 5 years compared to the control group over 5 years. In the study group, values were higher in the <5 years age group compared to the >5 years age group, with overall higher concentration values in the study group compared to the control group. However, these changes were statistically insignificant, with *p* = 0.701 (Table S2). Although these findings had no statistical significance, being under 5 years old may influence the levels of IL-6 due to the increased immune response in younger ages. Figure 2 shows the distribution of IL-6 values according to age in the control group and the study group.

#### 3.1.2. Comparisons of Serum Levels of Interleukin 6 (IL-6) by Sex in the Study Group and the Control Group

To analyze the serum levels of IL-6 by sex in the control group and the study group, we used the Kruskal–Wallis test for multiple comparisons because the criteria for normal distribution were not met.

**Figure 3:**
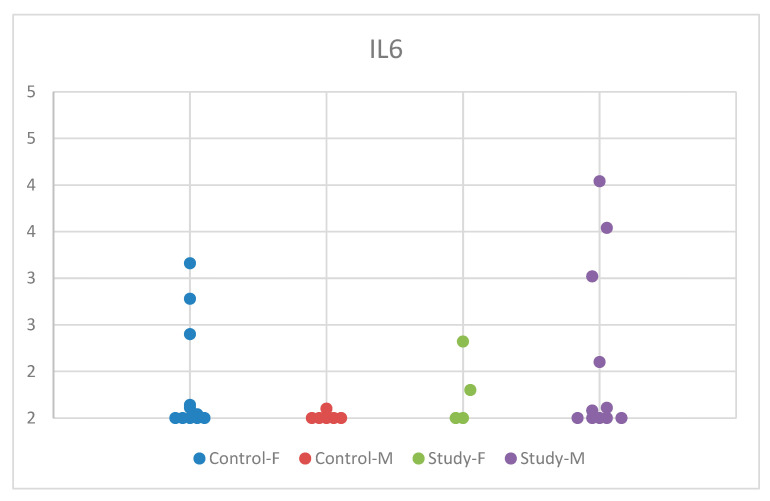
Scatter-plot of interleukin 6 (IL-6) concentrations in the study group and the control group by sex. There were four groups: Control-F = control group female, Control-M = control group male, Study-F = study group female, Study-M = study group male.

The descriptive analysis based on sex revealed statistically insignificant results, with *p* = 0.315 (Table S3). The distribution of IL-6 concentrations by sex in the study group and control group is comprehensively presented in Figure 3.

Interestingly, while the control group exhibited significantly lower serum IL-6 concentrations in boys compared to girls, autistic girls had marginally lower values compared to boys, albeit not statistically significant. In contrast, the serum IL-6 concentration in autistic girls was lower compared to controls, whereas in autistic boys, it was higher compared to controls. This may suggest sex-specific pathways regarding the development of ASD symptoms.

### 3.2. Results for Interleukin 8 (CXCL8). Descriptive Analysis, and Comparisons by Age and Sex between the Study Group and the Control Group

**Figure 4:**
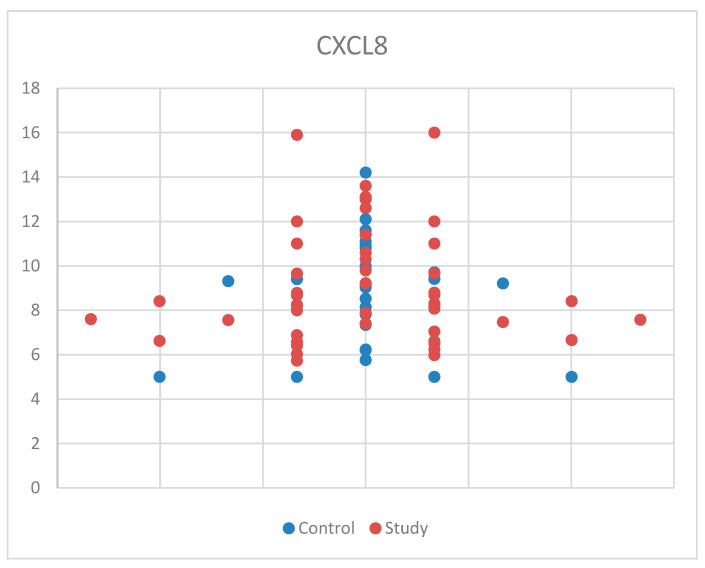
Scatter-plot of interleukin 8 (CXCL8) concentrations in the study group and the control group.

For CXCL8, we used Student’s *t*-test for equal variances because we met the normal distribution criteria and found no statistical significance between the study and control groups, with *p* = 0.343 (Table S1). Also, the median (and interquartile range) was 8.41 (6.77–10.15), with 8.78 (6.50–9.69) for the control group and 8.41 (7.04–10.60) for the study group (Table S4). For a better understanding of the CXCL8 concentrations between the study group and the control group, the data are presented in Figure 4. These results may be due to the relatively small sample size and the possibility that CXCL8 is not as strongly associated with ASD as hypothesized.

#### 3.2.1. Comparisons of Serum Levels of Interleukin 8 (CXCL8) by Age in the Study Group and the Control Group

**Figure 5:**
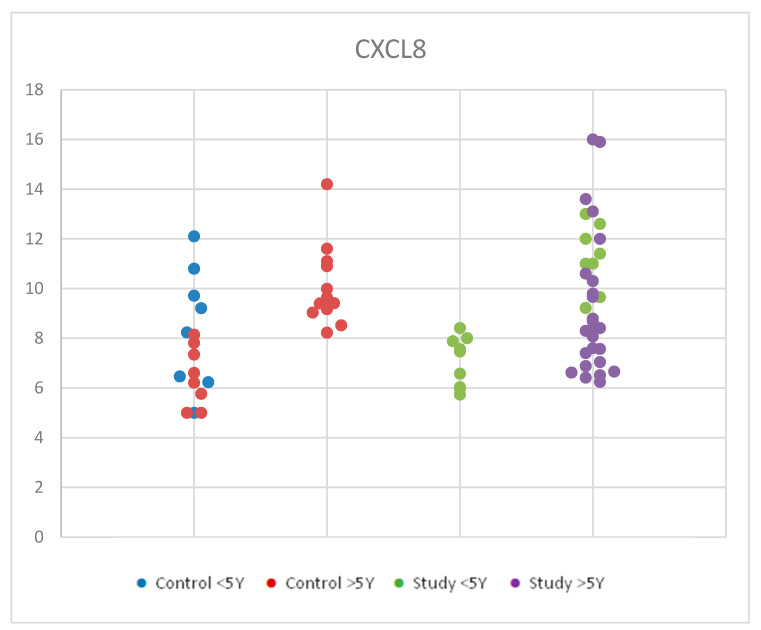
Scatter-plot of interleukin 8 (CXCL8) concentrations in the study group and the control group by age between 2 and 12 years. There were four groups: Control <5 Y = control group under 5 years old, Control >5 Y = control group over 5 years old, Study <5 Y = study group under 5 years old, Study >5 Y = study group over 5 years old.

For CXCL8, we used ANOVA for multiple comparisons due to Gaussian distribution in order to compare serum levels of interleukin 8 (CXCL8) by age in the study group and control group We found similar average values for each age group considered. Statistical analysis of CXCL8 values according to age groups for both the study and control groups revealed no significant differences between groups, with *p* = 0.780 (Table S2). The small sample size and the biological variety may be responsible for this outcome. Figure 5 shows the distribution of CXCL8 values according to age in the control group and the study group.

#### 3.2.2. Comparisons of Serum Levels of Interleukin 8 (CXCL8) by Sex in the Study Group and the Control Group

For CXCL8, we employed the ANOVA test for equal variances, because the normality criteria were met, and the Tukey test for multiple comparisons. The statistical analysis based on sex showed insignificant results, with *p* = 0.468 (Table S3).

**Figure 6:**
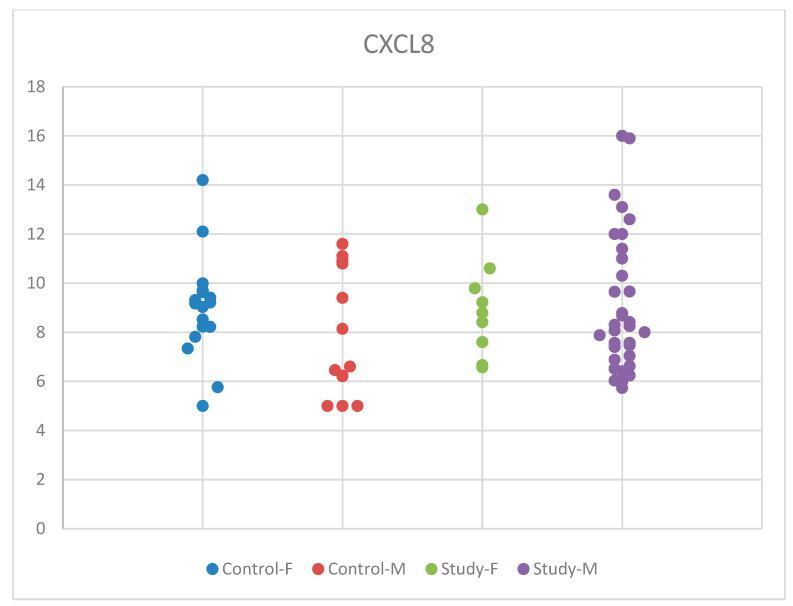
Scatter-plot of interleukin 8 (CXCL8) concentrations in the study group and the control group by sex. There were four groups: Control-F = control group female, Control-M = control group male, Study-F = study group female, Study-M = study group male.

Although mean serum CXCL8 concentrations were slightly higher in males than in females, with an increase observed in the group of children with autism compared to controls in boys and a slight decrease in the autism group compared to controls for girls, these changes were statistically insignificant. These differences according to sex, in the males’ favor, show that there might be differences in boys and girls regarding the immune response. Our results may be influenced by the small sample size of the groups that we studied. The distribution of CXCL8 concentrations by sex in the study and control groups is presented in Figure 6.

### 3.3. Results for Tumor Necrosis Factor Alpha (TNF-Alpha). Descriptive Analysis, and Comparisons by Age and Sex between the Study Group and the Control Group

**Figure 7:**
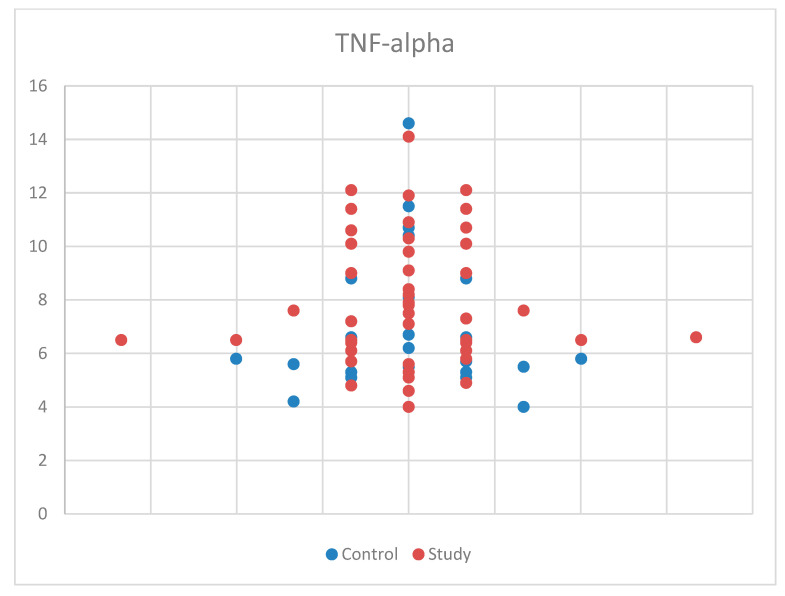
Scatter-plot of tumor necrosis factor alpha (TNF-alpha) concentrations in the study group and the control group.

For TNF-alpha meeting the normality criteria, we again used Student’s *t*-test for equal variances for comparisons by age and sex between the study group and the control group. We found no statistically significant results, with *p* = 0.100 (Table S1). Also, the median (and interquartile range) was: 7.10 (5.70–9.05), with 6.40 (5.50–8.05) for the control group and 7.50 (6.40–10.10) for the study group (Table S4). The sample size may have influenced the results for TNF-alpha. The distribution of TNF-alpha concentrations by sex in the study and control groups is presented in Figure 7.

#### 3.3.1. Comparisons of Serum Levels of Tumor Necrosis Factor Alpha (TNF-Alpha) by Age in the Study Group and the Control Group

**Figure 8:**
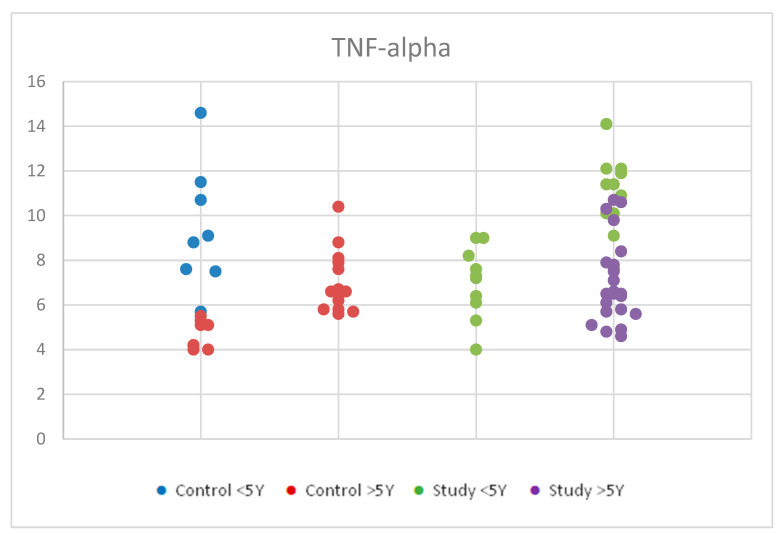
Scatter-plot of tumor necrosis factor alpha (TNF-alpha) concentrations in the study group and the control group by age between 2 and 12 years. There were four groups: Control <5 Y = control group under 5 years old, Control >5 Y = control group over 5 years old, Study <5 Y = study group under 5 years old, Study >5 Y = study group over 5 years old.

For TNF-alpha, we applied ANOVA for Gaussian distribution in order to compare the serum levels of tumor necrosis factor alpha (TNF-alpha) by age in the study group and control group. We observed statistically significant differences between the groups, with a *p*-value < 0.001 (Table S2). Figure 8 represents TNF-alpha concentrations in the study group and the control group by age.

Further statistical analysis of the mean values of TNF-alpha serum concentrations within the autism and control groups revealed significant differences between age groups using the Tukey HSD test (significance *p* < 0.05).

Children in both the control and study groups aged <5 years had higher TNF-alpha values than those >5 years. Specifically, within the groups, the study group <5 years had higher values than the study group >5 years (*p* = 0.007). Similarly, the control group < 5 years had higher values than the control group >5 years (*p* = 0.002).

Notably, there were false positive results observed between the study group <5 years and the control group >5 years (*p* < 0.001), and between the control group <5 years and the autism group >5 years (*p* = 0.032), possibly due to the low number of participants and their distribution within the age groups, which may have influenced the results.

Within the autism group, a Student’s *t*-test for equal variances showed statistical significance, indicating that children with autism aged <5 years had higher TNF-alpha values than those >5 years.

Given the increased values observed only for TNF-alpha, we focused our statistical analysis specifically on this cytokine and examined both qualitative and quantitative differences within the two age groups.

To assess the qualitative correlation between TNF-alpha levels (elevated/normal) and age groups (>5 years/<5 years), we applied the chi-square (X2) test.

Within the study group aged <5 years, a higher percentage of children exhibited high TNF-alpha values compared to normal values.

In contrast, within the study group aged >5 years, the majority of children had normal values. The same trend was observed in the control group (Table 2).

Overall, 40% of the children in the study group had high levels of TNF-alpha, while 60% had normal levels.

In the control group, 23.33% of children had high TNF-alpha levels, and 76.66% had normal levels.

The chi-square (χ^2^) test results indicate a significant correlation between TNF-alpha levels and age groups: χ^2^ (df = 1, *n* = 45) = 9.375, *p* = 0.005.

The relative risk for elevated TNF-alpha values is 3.250 times higher in the <5-year-old group compared to the >5-year-old group for children in the study group.

In the control group, the relative risk for elevated TNF-alpha is 2.499 for the <5-year-old group compared to the >5-year-old group, which is slightly lower than that in the study group.

#### 3.3.2. Comparisons of Serum Levels of Tumor Necrosis Factor Alpha (TNF-Alpha) by Sex in the Study Group and the Control Group

**Figure 9:**
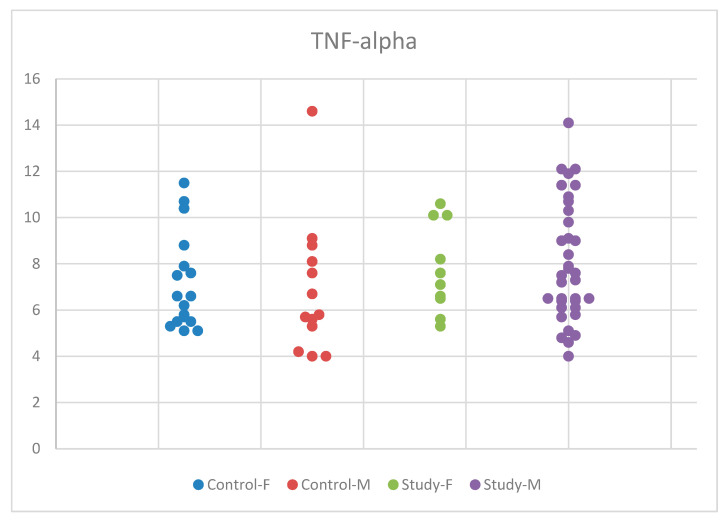
Scatter-plot of tumor necrosis factor alpha (TNF-alpha) concentrations in the study group and the control group by sex. There were four groups: Control-F = control group female, Control-M = control group male, Study-F = study group female, Study-M = study group male.

Regarding TNF-alpha, we applied ANOVA for equal variances and normal distribution. However, we did not find statistically significant results, with *p* = 0.428 (Table S3). The distribution of the IL-6 concentrations by sex in the study and control groups is presented in Figure 9.

Despite the increase in mean serum TNF-alpha concentrations in autistic boys compared to girls and the control group, the differences were not statistically significant. We conducted the Tukey HSD test for multiple comparisons between the concentrations obtained for TNF-alpha in the two participant groups, the study and control groups, and between the male and female sexes, but we did not identify statistically significant values.

### 3.4. Correlations between Increased Levels of TNF-Alpha and ASD Symptoms

We conducted statistical analyses to examine the correlations between TNF-alpha levels and ASD symptoms, as measured by the SCQ and ASRS scales. Our goal was to determine if high levels of TNF-alpha influenced ASD symptoms.

For TNF-alpha and SCQ, we applied the Pearson correlation due to the approximately normal distribution of the data. The results showed no statistical significance.

**Figure 10:**
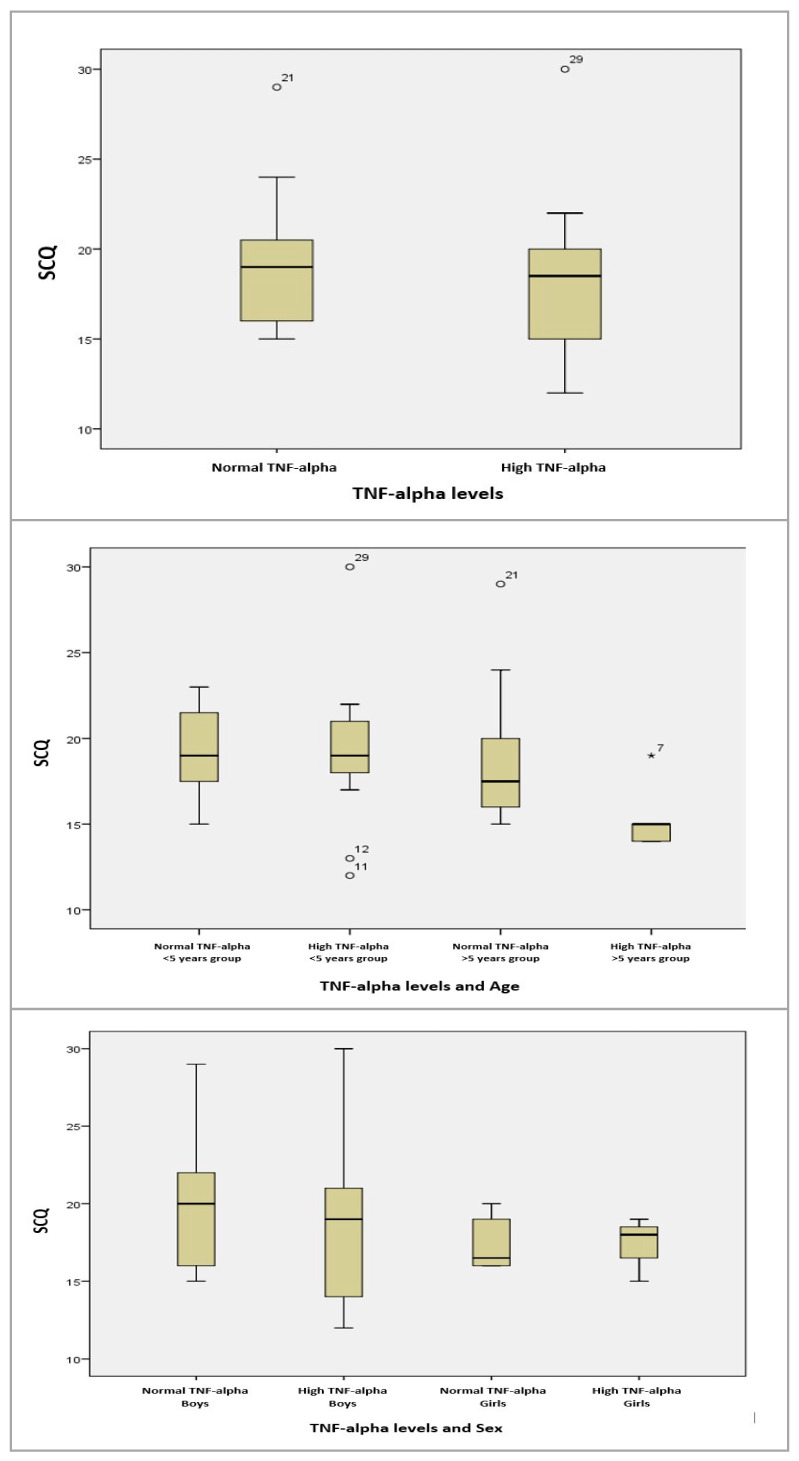
Boxplot graphs for serum TNF-alpha levels, and TNF-alpha levels depending on age, sex, and SCQ results. The circles and asterisk represent cases with extreme values outside the 95% CI (outliers). Between-group comparison was performed excluding outliers, and the computed statistical significances were observed without using outlier cases.

For the age groups (>5 years/<5 years) and SCQ, we conducted an ANOVA test for equal variances and found no statistically significant correlations. Similarly, for gender (male/female) and SCQ, the results were statistically insignificant (Figure 10).

Next, we explored the correlations between the results obtained on the ASRS qualitative scale and TNF-alpha concentrations in the study group. The ASRS scale includes social communication (SC) and unusual behaviors (UB) for two age groups (2–5 years and 6–18 years), self-regulation (SR), the DSM-IV-TR scale (DSM), and treatment scales: socialization with children of the same age (PS), socializing with adults (AS), social/emotional reciprocity (SER), atypical language (AL), stereotypes (ST), behavioral rigidity (BR), sensory sensitivity (SS), and attention/self-regulation (ASR).

**Chart 1:**
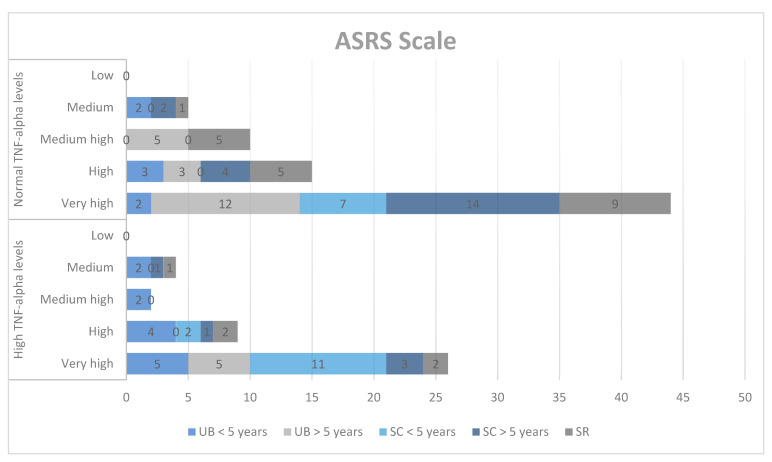
Qualitative analysis of the ASRS scale and TNF-alpha levels. UB = unusual behaviors, SC = social communication, SR = self-regulation.

**Chart 2:**
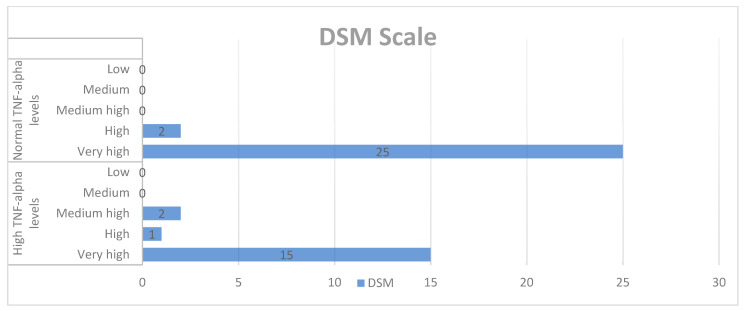
Qualitative analysis of the DSM-IV-TR scale and TNF-alpha levels.

**Chart 3:**
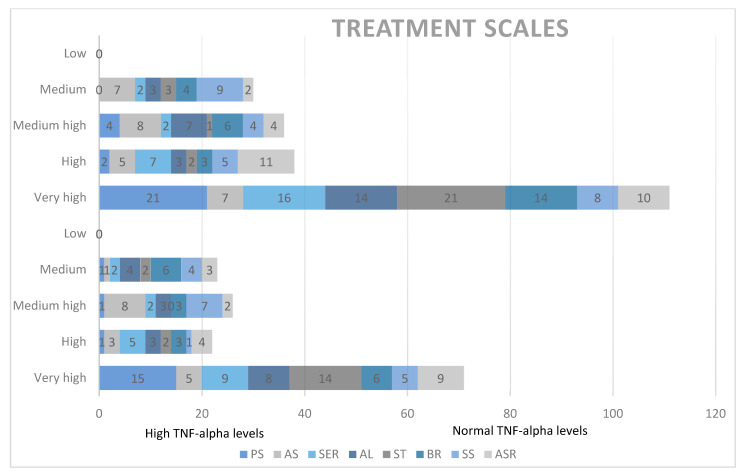
Qualitative analysis of treatment scales and TNF-alpha levels. PS = socialization with children of the same age, AS = socializing with adults, SER = social/emotional reciprocity, AL = atypical language, ST = stereotypes, BR = behavioral rigidity, SS = sensory sensitivity (SS), ASR = attention/self-regulation.

In the qualitative analysis, although we obtained high scores for SC, DSM, PS, ST, BR, and ASR, we did not find significant correlations between ASD symptoms and TNF-alpha levels (Chart 1, Chart 2 and Chart 3).

We considered the two different age groups (2–5 years and 6–18 years) for social communication (SC) and unusual behaviors (UB) specifically because the subscales in the ASRS are designed to account for developmental differences in younger and older children. These age-specific distinctions are crucial for accurately capturing the variations in social communication and unusual behaviors that occur as children grow older. For other parameters, such as self-regulation (SR), DSM-IV-TR scale (DSM), and treatment scales (PS, AS, SER, AL, ST, BR, SS, ASR), we considered the entire study group because these parameters are not as heavily influenced by developmental stages and can be more uniformly applied across the broader age range.

For the SC scale for children aged 2–5 years, we found no significant correlations. Similarly, for the SC scale for children aged 6–12 years, no correlations were observed. For the UB scale, no significant correlations were found for either age group.

When examining the correlation between the DSM scale and TNF-alpha concentrations in the study group, no statistical significance was found.

Additionally, correlations between treatment scales and TNF-alpha concentrations in the study group yielded non-significant results.

These findings, pertaining to the correlations between ASRS scales and subscales and TNF-alpha serum concentrations, were not statistically significant. In summary, our analysis revealed no significant correlations between elevated serum TNF-alpha levels and ASD symptomatology. Although TNF-alpha was related to autism symptoms in previous studies, in our study, the lack of significance between the levels of TNF-alpha may be due to sample size limitation, slight differences regarding sex distributions, and possibly biological variability.

## 4. Discussion

A growing body of research suggests that brain inflammation, particularly during perinatal development, can induce neurodegenerative changes. These changes affect critical areas such as the amygdala, which is crucial for emotional processing and fear regulation. Various factors, including neuropeptides, stress, toxins, and viruses, can trigger brain inflammation by activating mast cells. This activation leads to microglial activation [28]. The resulting inflammatory cascade involves monocytes, macrophages, mast cells, and microglia. This process, combined with increased production of proinflammatory cytokines, contributes to neuroinflammation. Neuroinflammation has been implicated in the onset and progression of ASD [29]. Maternal immune activation (MIA) can also drive inflammation, resulting in elevated cytokine levels like IL-6, IL-17A, and TNF-alpha. These cytokines can cross the placenta and affect fetal development [30]. Although abnormal cytokine levels in autism are well-documented, the precise mechanisms underlying these alterations remain elusive [31].

### 4.1. Mechanistic Pathways of Cytokines in Neurodevelopment and ASD

Cytokines are critical mediators in the communication between the immune system and the central nervous system (CNS). They play a significant role in neurodevelopment and behavior, especially through their influence on synaptic regulation, neuroplasticity, and neurotransmitter systems. The mechanisms by which cytokines like IL-6 and TNF-alpha contribute to synaptic dysregulation and ASD-like symptoms can be understood through several key pathways.

### 4.2. IL-6 and Its Dual Role in Neurodevelopment

IL-6 is a multifunctional cytokine with both pro-inflammatory and anti-inflammatory properties. It influences various cell types, including neurons, glial cells, and endothelial cells. These effects are crucial for processes such as cell growth, differentiation, and survival. The dual role of IL-6 in inflammation and neuroprotection is central to understanding its impact on the brain.

Pro-inflammatory effects: IL-6 is produced rapidly in response to infections, trauma, and stress. It triggers acute phase responses and enhances the production of other pro-inflammatory cytokines. During neurodevelopment, elevated IL-6 levels can disrupt the delicate balance of neuronal and synaptic development. IL-6 can alter synaptic plasticity by changing the expression of synaptic proteins and neurotransmitter receptors. This alteration can lead to abnormal synapse formation and function, which are characteristic of ASD [12,32].

Impact on neurogenesis and synaptogenesis: IL-6 can influence the differentiation of neural stem cells and the maturation of neurons. Chronic elevation of IL-6, as seen in MIA, can lead to aberrant synaptogenesis. This condition is linked to the behavioral and cognitive deficits observed in ASD. Animal models have shown that IL-6 can alter the expression of genes involved in synaptic transmission and plasticity, contributing to the synaptic abnormalities associated with ASD [29,33].

Crossing the placenta: IL-6 can cross the placenta and influence fetal brain development by promoting local inflammation within the fetal brain. This inflammatory environment can disrupt normal neurodevelopmental processes, leading to long-term changes in brain structure and function. These changes may manifest as ASD-like behaviors later in life [29,34].

In our study, we did not observe significant correlations between IL-6 levels and ASD within our study groups. However, we noted a slight increase in serum concentration values from the control group under 5 years old compared to the group over 5 years old. In the study group, values were higher in the under-5 years of age group compared to the over-5 years of age group. Overall, the mean serum concentrations were higher in the study group compared to the control group, though these changes were statistically insignificant. Notably, while the control group exhibited significantly lower serum IL-6 concentrations in boys compared to girls, the study group showed marginally lower values in girls compared to boys. Autistic girls displayed lower IL-6 levels compared to controls, whereas autistic boys exhibited higher levels compared to controls.

Although we did not observe statistically significant correlations between IL-6 levels and ASD, the observed trends suggest potential biological relevance. Several studies, such as those by Ashwood et al. [15], have reported significant associations between elevated IL-6 levels and more severe ASD symptoms, particularly in the context of stereotypies and repetitive behaviors. Our findings, while not statistically significant, align with the notion that IL-6 may play a role in ASD, particularly in subgroups of individuals. The discrepancies between our results and those of previous studies could be due to differences in sample size, population heterogeneity, or the timing of cytokine measurement. The slight elevation in IL-6 among boys with ASD compared to girls observed in our study could suggest sex-specific pathways that contribute to ASD, which merits further investigation. Future studies could explore these trends by focusing on specific ASD subtypes or by examining IL-6 in conjunction with other biomarkers to identify potential interactions.

### 4.3. TNF-Alpha and Its Role in Neuroinflammation

TNF-alpha is a potent pro-inflammatory cytokine with significant effects on the CNS. It plays a key role in regulating immune responses, apoptosis, and synaptic plasticity. TNF-alpha’s role in neurodevelopment is particularly critical in the context of neuroinflammation.

Synaptic modulation: TNF-alpha regulates synaptic scaling, a process that adjusts the strength of synapses to maintain overall network stability. However, excessive TNF-alpha can lead to dysregulation of synaptic scaling. This dysregulation results in either hyper- or hypo-excitability of neural circuits, which is thought to contribute to the synaptic abnormalities observed in ASD. These abnormalities include altered connectivity and impaired communication between neurons [35,36,37].

Microglial activation: TNF-alpha can activate microglia, the resident immune cells of the CNS, leading to sustained inflammation within the brain. Activated microglia release additional cytokines and neurotoxic factors, which can exacerbate neuronal damage and disrupt synaptic function. Chronic microglial activation is implicated in various neurodevelopmental disorders, including ASD, where it may contribute to neuroinflammation and synaptic deficits [29,35].

Impact on neurodevelopment: During critical periods of neurodevelopment, TNF-alpha influences processes such as neuronal migration, differentiation, and axonal growth. Elevated levels of TNF-alpha during these periods, as seen in MIA, can alter brain architecture and connectivity. This alteration can lead to the neurodevelopmental abnormalities characteristic of ASD. Additionally, TNF-alpha can disrupt the blood–brain barrier, allowing peripheral immune cells and cytokines to enter the brain. This entry further contributes to neuroinflammation and neurodevelopmental disorders [35,38,39,40,41,42].

TNF-alpha does not act in isolation but is part of a complex cytokine network that interacts to shape the neuroinflammatory environment observed in ASD. For instance, TNF-alpha works synergistically with other pro-inflammatory cytokines like IL-1β and IL-6 to amplify inflammatory responses. These cytokines can collectively influence synaptic plasticity by modulating the expression of neurotransmitter receptors and synaptic proteins, leading to altered synaptic strength and connectivity, which are hallmarks of ASD. Moreover, the interaction between TNF-alpha and anti-inflammatory cytokines, such as IL-10, is crucial in maintaining the balance between neuroinflammation and neuroprotection. Disruptions in this balance, as observed in ASD, can exacerbate neuronal dysfunction and contribute to the persistence of ASD symptoms.

Furthermore, the role of TNF-alpha in activating microglia is particularly significant in the context of chronic neuroinflammation. Microglia, once activated, can produce a range of pro-inflammatory cytokines, including TNF-alpha, IL-1β, and IL-6, creating a self-perpetuating cycle of inflammation that may lead to long-term changes in brain function and structure. This persistent inflammatory state can interfere with normal processes of synaptic pruning and plasticity, which are critical during early brain development, potentially leading to the neurodevelopmental deficits observed in ASD.

In summary, TNF-alpha, in conjunction with other cytokines, contributes to a complex network that drives neuroinflammation and synaptic dysregulation in ASD. Understanding the interplay between these cytokines and their collective impact on neurodevelopment is crucial for identifying potential therapeutic targets aimed at modulating neuroinflammatory pathways and mitigating ASD symptoms.

In our study, we observed significant results regarding TNF-alpha levels. Children under 5 years of age exhibited higher TNF-alpha values compared to those over 5 years old. Specifically, 40% of children with autism under 5 years had elevated TNF-alpha levels compared to their older counterparts. The relative risk for high TNF-alpha values was notably higher in the under-5 years of age group, being 3.250 times higher than in the over-5 years of age group. These findings suggest the potential involvement of TNF-alpha in the inflammatory processes associated with autism symptoms.

Our findings regarding TNF-alpha are consistent with several studies that have demonstrated elevated TNF-alpha levels in children with ASD, particularly those with more severe symptoms or comorbid conditions such as gastrointestinal disturbances [42,43]. For example, studies by Vargas et al. [44] and Li et al. [45] have reported significant correlations between TNF-alpha levels and ASD severity. The elevated TNF-alpha levels in younger children in our study might indicate a more pronounced inflammatory response during early development, which could contribute to the onset and progression of ASD symptoms. However, some studies, such as those by Goines et al. [46], have not found significant differences in TNF-alpha levels between ASD and control groups, suggesting that TNF-alpha’s role in ASD may vary across different populations or subtypes. The methodological differences, including sample size, timing of sample collection, and population characteristics, could account for these discrepancies. Further research is needed to clarify TNF-alpha’s role in ASD and to explore whether it could serve as a biomarker for early diagnosis or a target for therapeutic intervention.

### 4.4. Cytokine Interactions and Neurodevelopmental Outcomes

The interactions between IL-6, TNF-alpha, and other cytokines create a complex inflammatory environment that profoundly impacts neurodevelopment. The balance between pro-inflammatory and anti-inflammatory cytokines is crucial in determining the outcome of immune activation in the developing brain. Disruptions in this balance, such as those caused by MIA, can lead to long-lasting changes in brain function and structure. These changes predispose individuals to ASD and other neurodevelopmental disorders.

In summary, the mechanistic pathways involving IL-6 and TNF-alpha highlight their critical roles in synaptic dysregulation and neuroinflammation. These factors are central to the pathophysiology of ASD. By influencing synaptic plasticity, neuronal survival, and microglial activity, these cytokines contribute to the neurodevelopmental abnormalities and behavioral symptoms observed in ASD. Understanding these mechanisms provides valuable insights into potential therapeutic targets for modulating cytokine activity in the treatment of ASD.

In our study, we examined serum concentrations of interleukin 8 (CXCL8), IL-6, and tumor necrosis factor alpha (TNF-alpha) to explore potential links between immune profiles and ASD compared to neurotypical controls.

CXCL8: CXCL8, also known as IL-8, plays a crucial role in inflammation by recruiting neutrophils, basophils, and certain T lymphocytes to sites of injury [47,48,49]. Elevated CXCL8 levels in peripheral blood often coincide with reduced neutrophil counts at inflammatory sites and systemic neutrophilia [50,51]. Recent studies, such as Shen Y. et al.’s 2021 research, propose CXCL8 as a promising ASD biomarker, potentially involved in ASD pathogenesis [52]. Interestingly, CXCL8 has been associated with parental cytokine levels and specific subdomains of the Social Responsiveness Scale (SRS) in children with ASD [53]. Although few studies have explored parental cytokine levels, elevated CXCL8 plasma levels have been linked to ASD traits like stereotypies, hyperactivity, and lethargy [12,53].

In our investigation, we did not observe significant correlations between CXCL8 levels and ASD. However, while statistically insignificant, average CXCL8 concentrations tended to be slightly higher in the autistic group compared to the control group across age groups, with slightly elevated levels in boys compared to girls.

TNF-alpha: For TNF-alpha, our findings revealed significant results that suggest the potential involvement of TNF-alpha in the inflammatory processes associated with autism symptoms.

During childhood, innate immune systems tend to be more active compared to adults, undergoing maturation processes to regulate themselves [54,55]. However, the child’s immune system may struggle to adapt to interactions among immune factors, leading to responses to adverse factors that could affect the nervous system via increases in pro-inflammatory cytokines [56,57].

TNF-alpha, a pro-inflammatory cytokine, is designed to destroy tumor cells by activating protein transcription upon binding to its receptors. This leads to cell proliferation, inflammatory responses, and apoptosis. TNF-alpha is expressed by various cell types including macrophages, monocytes, lymphocytes, and astrocytes. It influences synaptogenesis and microglial cell activity, potentially impacting neurodevelopment [35,36,37]. TNF-alpha also plays a crucial role in inducing placental pathology, affecting fetal brain development [38,39,40,41,42].

Studies have reported increased levels of TNF-alpha in children with autism, particularly those with gastrointestinal symptoms. This suggests a potential therapeutic pathway for addressing both symptoms [43]. While some studies found no significant differences in TNF-alpha levels between autism and control groups [54], others demonstrated that interventions targeting TNF-alpha, such as valproic acid exposure in mice, could reduce neuroinflammation and improve behavioral outcomes [42,55].

Disruptions in the cytokine network, including increased TNF-alpha levels, have been associated with neurodevelopmental abnormalities and behavioral symptoms in autism [56,57]. Notably, some children with autism show behavioral improvements during fever-induced inflammation, highlighting the potential role of TNF-alpha in autism severity [58,59].

In a recent meta-analysis, abnormal levels of various cytokines in the peripheral blood of ASD patients compared to controls were identified. These include IL-6, IL-1β, IL-12p70, MIF, eotaxin-1, MCP-1, CXCL8, IL-7, IL-2, IL-12, TNF-alpha, IL-17, and IL-4 [60]. These abnormal cytokine profiles hold promise as potential biomarkers for ASD diagnosis and treatment [61].

Cao et al. found elevated levels of IL-6, IL-17, TNF-alpha, and IL-1β in the plasma and serum of autistic subjects compared to controls [31]. They identified monocytes, neutrophils, and CD4+ T cells as potential sources of these elevated cytokines in autism. Additionally, cytokines such as IFN-γ, TGF-β, RANTES, and CXCL8 were also elevated in the plasma/serum of subjects with autism, with IFN-γ likely produced by CD4+ T cells and natural killer (NK) cells. However, there is conflicting evidence regarding IFN-γ and TGF-β [31]. Conversely, cytokines like IL-13, IL-10, IL-5, and IL-4 were found to be unaltered in plasma/serum and post-stimulated blood immune cells in autistic individuals compared to controls [31].

TNF-alpha, a ligand for the ErbB signaling pathway, was found to be increased in the postmortem brain and serum of ASD cases compared to healthy controls [61]. Despite consistent reports of abnormal circulating blood cytokine levels in autism, the underlying mechanisms driving these changes remain unclear [31]. Therefore, we sought to investigate potential correlations between TNF-alpha levels and SCQ and ASRS test results in our study group. However, we did not observe statistically significant results, indicating a lack of relationship between TNF-alpha levels and the severity of ASD symptoms in our study.

## 5. Limitations and Strengths

While our study offers valuable insights into the role of cytokines in autism spectrum disorder (ASD), it is important to consider certain limitations that could influence the interpretation of our findings. Despite these limitations, the study remains robust and contributes meaningfully to the understanding of ASD, particularly in early childhood.

### 5.1. Sample Size and Statistical Power

One limitation of our study is the relatively small sample size, which may impact the generalizability of the findings and reduce the statistical power to detect subtle effects. However, even with this sample size, we were able to identify significant trends and associations, particularly concerning TNF-alpha levels in younger children with ASD. A larger sample could enhance these findings, providing even greater confidence in the observed patterns.

### 5.2. Selection Bias and Population Specificity

The recruitment of participants from a specific medical office may introduce some degree of selection bias, potentially limiting the representativeness of the sample. Nonetheless, the study’s focus on a clinically relevant population—children actively seeking evaluation for ASD—adds value by closely mirroring the conditions under which ASD is typically diagnosed and treated. This focus enhances the clinical relevance of our findings, even if it may limit broader generalizability.

### 5.3. Gender Distribution and Its Impact

While there were discrepancies in gender distribution within our sample, this aspect also highlights an important consideration for future research. The gender differences observed in cytokine levels and ASD symptoms underscore the need for more nuanced studies that specifically explore sex-based biological differences in ASD. Our findings contribute to this growing area of research, suggesting potential pathways that merit further investigation.

### 5.4. Dietary and Environmental Factors

Although we could not control for dietary differences among participants, the study’s findings still provide a strong foundation for understanding the role of cytokines in ASD. Future studies could build on this work by incorporating more detailed dietary and environmental assessments, which would further elucidate the complex interactions between these factors and immune function in ASD.

### 5.5. Control-Group Recruitment

Recruiting a control group posed challenges, yet we succeeded in assembling a comparison group that, while not perfect, still allowed for meaningful contrasts with the ASD group. The results from this comparison contribute valuable insights into the cytokine profiles associated with ASD, and they highlight the importance of continuing to refine control-group selection in future research.

### 5.6. Lack of Longitudinal Data

While our study captured data at a single time point, it provides an important snapshot of cytokine levels during a critical developmental window. This cross-sectional approach lays the groundwork for future longitudinal studies, which could further explore how cytokine levels and ASD symptoms evolve over time, potentially leading to more dynamic models of ASD pathophysiology.

### 5.7. Single-Center Study and Generalizability

Conducting the study at a single center may limit its generalizability to other populations. However, this approach also allowed for a controlled and consistent methodology, ensuring the reliability of our data. Multicenter studies in the future could expand on these findings, helping to validate and extend the conclusions drawn here across diverse populations.

### 5.8. Strengths

Despite these limitations, our study has several key strengths that enhance its contribution to the field. By focusing on early childhood, we provide insights into the developmental stages of ASD that are less frequently studied but critically important for early intervention. The comprehensive analysis of multiple cytokines, including IL-6, CXCL8, and TNF-alpha, offers a broad perspective on the immune dysregulation potentially involved in ASD. Furthermore, our use of rigorous statistical methods, including multiple tests and post hoc analyses, adds robustness to our findings, ensuring that they are both reliable and meaningful.

## 6. Future Directions

Building on the findings of our study, future research should aim to refine and expand upon our results through the following methodological enhancements:

Larger and more diverse cohorts: Future studies should include larger sample sizes with a diverse demographic composition, including participants from various socioeconomic backgrounds and ethnicities. This would help validate our preliminary findings and ensure that they are robust and generalizable across different populations. Larger cohorts would also increase the statistical power of the study, enabling the detection of smaller yet potentially significant effects.

Longitudinal study designs: Employing longitudinal designs is crucial to track changes in cytokine levels and ASD symptoms over time. This approach would provide a clearer understanding of the temporal relationship between immune dysregulation and the progression of ASD. Longitudinal studies could also identify critical periods during which cytokine levels may have the most significant impact on neurodevelopment.

Multi-center studies: Expanding the research to include multiple centers across various geographic locations would help address the limitations associated with single-center studies. This would improve the generalizability of the findings and allow for the examination of regional variations in cytokine levels and ASD prevalence. Collaboration between institutions could also facilitate larger, more diverse sample collections.

Balanced gender representation: Ensuring a balanced representation of male and female participants in future studies is essential for clarifying the role of gender in cytokine levels and ASD symptomatology. This would help to identify any sex-specific differences that could influence the development and manifestation of ASD, potentially leading to more tailored interventions.

Control for confounding variables: Future research should rigorously control for potential confounding variables, such as dietary habits, recent infections, sleep patterns, and environmental exposures. Incorporating these controls would help isolate the effects of cytokines on ASD from other influencing factors, providing a more accurate assessment of their roles in the disorder.

Inclusion of additional cytokines: Future studies should consider including a broader range of cytokines, such as IL-10, IL-17, and others that are implicated in immune regulation and inflammation. This would provide a more comprehensive immune profile of children with ASD, potentially identifying additional biomarkers or therapeutic targets.

Advanced statistical analyses: To better understand the complex relationships between cytokine levels, ASD symptoms, and various confounding factors, future research should employ advanced statistical methods. Generalized linear models (GLMs), multivariate analyses, and machine-learning techniques could help disentangle these relationships and identify patterns that are not apparent through traditional statistical approaches. These methods would also allow for the integration of multiple variables and the assessment of their combined effects on ASD.

By adopting these methodological improvements, future research can build on our findings and contribute to a deeper understanding of the role of cytokines in ASD, ultimately guiding more effective interventions and treatments.

## 7. Conclusions

In summary, our study yielded significant findings regarding TNF-alpha levels, which were elevated in some children diagnosed with ASD as well as in some children without psychiatric pathology. Notably, children under 5 years of age exhibited significantly higher TNF-alpha levels, likely due to the immune system’s instability during early childhood. These findings suggest that TNF-alpha could serve as an early biomarker for ASD, particularly in younger children, which could have important implications for early diagnosis and intervention.

Although we found no correlations between CXCL8 levels and ASD, our observations suggest a trend toward slightly higher CXCL8 concentrations in the autism group, particularly among boys. Similarly, serum concentrations of IL-6 were elevated in boys with autism compared to controls, with higher values observed in the younger age subgroup. While these findings are statistically insignificant, they highlight the potential of IL-6 as a biomarker that warrants further investigation in larger studies, especially in the context of early childhood ASD. Early identification of such biomarkers could lead to more personalized and timely therapeutic approaches, potentially improving long-term outcomes for children with ASD.

The clinical relevance of our findings is particularly evident in the elevated relative risk for high TNF-alpha levels among ASD children under 5 years. This underscores TNF-alpha’s potential role not just as a marker of inflammation but as a critical component in the pathophysiological processes underlying ASD. The identification of TNF-alpha as a potential biomarker could influence clinical practice by providing a tangible target for early intervention strategies aimed at modulating immune responses in children at risk for or diagnosed with ASD.

Further research with larger cohorts is warranted to conclusively determine the implications of CXCL8, IL-6, and TNF-alpha in early childhood autism. By identifying metabolic biomarkers, we aim to enhance the accuracy of ASD diagnosis, enabling personalized therapeutic approaches. However, it is crucial to interpret these findings cautiously due to the current divergent nature of the literature.

In conclusion, our study not only contributes to the understanding of ASD biomarkers but also underscores their potential clinical utility. By supporting the establishment of etiology, diagnosis, and treatment planning, these biomarkers could play a pivotal role in enhancing the quality of life for individuals with ASD. The possibility of integrating TNF-alpha and potentially IL-6 into routine clinical practice could mark a significant advancement in the early detection and personalized management of ASD.

## Figures and Tables

**Table 1:** Distribution data for the study group and the control group.

Data Distribution	<5 Years Old (*n* = 40)	>5 Years Old (*n* = 35)	Boys (%)	Girls (%)	Urban Area (%)	Rural Area (%)
Study group (*n* = 45)	25	20	35 (77.777%)	10 (22.222%)	34 (75.555%)	11 (24.444%)
Control group (*n* = 30)	15	15	13 (43.333%)	17 (56.666%)	26 (86.666%)	4 (13.333%)
Mean age ± SD	20 ± 7.071	17.5 ± 3.535	-	-	-	-
*p*-value	0.355		-	-	-	-

*n* = number, SD = standard deviation, *p*-value, significant for *p* < 0.05, % = percent.

**Table 2:** Crosstabulation of age for TNF-alpha levels in the study group and the control group.

TNF-Alpha Levels	% Within Age Range			
	Study Group		Control Group	
	Age <5 Years	Age >5 Years	Age <5 Years	Age >5 Years
High	65.0% (*n* = 13)	20.0% (*n* = 5)	71.42% (*n* = 5)	28.57% (*n* = 2)
Normal	35.0% (*n* = 7)	80.0% (*n* = 20)	17.39% (*n* = 4)	82.60% (*n* = 19)

*n* = number of participants, % = percent.

## Data Availability

We confirm that the main data supporting the findings of this study are available within the article, and any other additional data are available on request.

## Supplementary Materials

The following supporting information can be downloaded at: https://www.mdpi.com/article/10.3390/life14091201/s1, Table S1: Means and standard deviations of serum interleukin 6 (IL-6), interleukin 8 (CXCL8), and tumor necrosis factor alpha (TNF-alpha) in the study group and the control group for ages between 2 and 12 years old. Table S2. Means and standard deviations of serum tumor necrosis factor alpha (TNF-alpha), interleukin 8 (CXCL8), and interleukin 6 (IL-6) concentrations by age in the study group and the control group. Table S3. Means and standard deviations of serum tumor necrosis factor alpha (TNF-alpha), interleukin 8 (CXCL8), and interleukin 6 (IL-6) concentrations by age in the study group and the control group. Table S4. Statistical analysis of interleukin 6, interleukin 8 and tumor necrosis factor alpha with mean, standard deviation, median, and interquartile range (IQR) results.

## References

[1] Hughes H., Moreno R.J., Ashwood P. (2023). Innate immune dysfunction and neuroinflammation in autism spectrum disorder (ASD). Brain Behav. Immun..

[2] Yirmiya R., Goshen I. (2011). Immune modulation of learning, memory, neural plasticity and neurogenesis. Brain Behav. Immun..

[3] Bourgognon J.-M., Cavanagh J. (2020). The role of cytokines in modulating learning and memory and brain plasticity. Brain Neurosci. Adv..

[4] Chen Y., Dai J., Tang L., Mikhailova T., Liang Q., Li M., Zhou J., Kopp R.F., Weickert C., Chen C. (2023). Neuroimmune transcriptome changes in patient brains of psychiatric and neurological disorders. Mol. Psychiatry.

[5] Capuron L., Miller A.H. (2011). Immune system to brain signaling: Neuropsychopharmacological implications. Pharmacol. Ther..

[6] Stenken J.A., Poschenrieder A.J. (2015). Bioanalytical chemistry of cytokines—A review. Anal. Chim. Acta..

[7] Mead J., Ashwood P. (2015). Evidence supporting an altered immune response in ASD. Immunol. Lett..

[8] Masi A., Glozier N., Dale R., Guastella A.J. (2017). The Immune System, Cytokines, and Biomarkers in Autism Spectrum Disorder. Neurosci. Bull..

[9] El-Ansary A., Al-Ayadhi L. (2014). GABAergic/glutamatergic imbalance relative to excessive neuroinflammation in autism spectrum disorders. J. Neuroinflamm..

[10] Obi-Nagata K., Temma Y., Hayashi-Takagi A. (2019). Synaptic functions and their disruption in schizophrenia: From clinical evidence to synaptic optogenetics in an animal model. Proc. Jpn. Acad. Ser. B Phys. Biol. Sci..

[11] Ahmad S.F., Ansari M.A., Nadeem A., Bakheet S.A., Al-Ayadhi L.Y., Alsaad A.M., Assiri M.A., Al-Mazroua H.A., Attia S.M. (2020). Upregulation of interleukin (IL)-31, a cytokine producing CXCR1 peripheral immune cells, contributes to the immune abnormalities of autism spectrum disorder. J. Neuroimmunol..

[12] Tanaka T., Narazaki M., Kishimoto T. (2014). IL-6 in inflammation, immunity, and disease. Cold Spring Harb. Perspect. Biol..

[13] Scheller J., Chalaris A., Schmidt-Arras D., Rose-John S. (2011). The pro- and anti-inflammatory properties of the cytokine interleukin-6. Biochim. Biophys. Acta.

[14] Garland E.F., Hartnell I.J., Boche D. (2022). Microglia and Astrocyte Function and Communication: What Do We Know in Humans?. Front. Neurosci. Sec. Neurodegener..

[15] Ashwood P., Krakowiak P., Hertz-Picciotto I., Hansen R., Pessah I., Van de Water J. (2011). Elevated plasma cytokines in autism spectrum disorders provide evidence of immune dysfunction and are associated with impaired behavioral outcome. Brain Behav. Immun..

[16] Mor G., Cardenas I. (2010). The Immune System in Pregnancy: A Unique Complexity. Am. J. Reprod. Immunol..

[17] Costa A.N., Ferguson B.J., Hawkins E., Coman A., Schauer J., Ramirez-Celis A., Hecht P.M., Bruce D., Tilley M., Talebizadeh Z. (2023). The Relationship between Maternal Antibodies to Fetal Brain and Prenatal Stress Exposure in Autism Spectrum Disorder. Metabolites.

[18] Kaminski V.L., Michita R.T., Ellwanger J.H., Veit T.D., Schuch J.B., Riesgo R.D.S., Roman T., Chies J.A.B. (2023). Exploring potential impacts of pregnancy-related maternal immune activation and extracellular vesicles on immune alterations observed in autism spectrum disorder. Heliyon.

[19] Nielsen T.C., Nassar N., Shand A.W., Jones H.F., Han V.X., Patel S., Guastella A.J., Dale R.C., Lain S.J. (2022). Association of maternal autoimmune disease and early childhood infections with offspring autism spectrum disorder: A population-based cohort study. Autism Res..

[20] Deckmann I., Schwingel G.B., Fontes-Dutra M., Bambini-Junior V., Gottfried C. (2018). Neuroimmune Alterations in Autism: A Translational Analysis Focusing on the Animal Model of Autism Induced by Prenatal Exposure to Valproic Acid. Neuroimmunomodulation.

[21] Sarieva K., Kagermeier T., Khakipoor S., Atay E., Yentür Z., Becker K., Mayer S. (2023). Human brain organoid model of maternal immune activation identifies radial glia cells as selectively vulnerable. Mol. Psychiatry.

[22] Majerczyk D., Ayad E.G., Brewton K.L., Saing P., Hart P.C. (2022). Systemic maternal inflammation promotes ASD via IL-6 and IFN-γ. Biosci. Rep..

[23] Zawadzka A., Cieślik M., Adamczyk A. (2021). The Role of Maternal Immune Activation in the Pathogenesis of Autism: A Review of the Evidence, Proposed Mechanisms and Implications for Treatment. Int. J. Mol. Sci..

[24] Liu X., Liu H., Gu N., Pei J., Lin X., Zhao W. (2023). Preeclampsia promotes autism in offspring via maternal inflammation and fetal NFkappaB signaling. Life Sci. Alliance.

[25] Liao X., Liu Y., Fu X., Li Y. (2020). Postmortem Studies of Neuro inflammation in Autism Spectrum Disorder: A Systematic Review. Mol. Neurobiol..

[26] Rutter M., Bailey A., Lord C. (2003). Social Communication Questionnaire (SCQ).

[27] Goldstein S., Naglieri J.A. (2009). Autism Spectrum Rating Scales (ASRS).

[28] Theoharides T.C. (2021). Ways to Address Perinatal Mast Cell Activation and Focal Brain Inflammation, including Response to SARS-CoV-2, in Autism Spectrum Disorder. J. Pers. Med..

[29] Lampiasi N., Bonaventura R., Deidda I., Zito F., Russo R. (2023). Inflammation and the Potential Implication of Macrophage-Microglia Polarization in Human ASD: An Overview. Int. J. Mol. Sci..

[30] Usui N., Kobayashi H., Shimada S. (2023). Neuroinflammation and Oxidative Stress in the Pathogenesis of Autism Spectrum Disorder. Int. J. Mol. Sci..

[31] Cao W., Luo C., Fan Z., Lei M., Cheng X., Shi Z., Mao F., Xu Q., Fu Z., Zhang Q. (2023). Analysis of potential biomarkers and immune infiltration in autism based on bioinformatics analysis. Medicine.

[32] Singh R., Kisku A., Kungumaraj H., Nagaraj V., Pal A., Kumar S., Sulakhiya K. (2023). Autism Spectrum Disorders: A Recent Update on Targeting Inflammatory Pathways with Natural Anti-Inflammatory Agents. Biomedicines.

[33] Filippova Y.Y., Devyatova E.V., Alekseeva A.S., Burmistrova A.L. (2022). Cytokines and neurotrophic factors in the severity assessment of children autism. Russ. Clin. Lab. Diagn..

[34] Theoharides T.C., Doyle R. (2008). Autism, Gut-Blood-Brain Barrier, and Mast Cells. J. Clin. Psychopharmacol..

[35] Jang D.-I., Lee A.-H., Shin H.-Y., Song H.-R., Park J.-H., Kang T.-B., Lee S.-R., Yang S.-H. (2021). The Role of Tumor Necrosis Factor Alpha (TNF-α) in Autoimmune Disease and Current TNF-α Inhibitors in Therapeutics. Int. J. Mol. Sci..

[36] Aldossari A.A., Ansari M.A., Nadeem A., Attia S.M., Bakheet S.A., Al-Ayadhi L.Y., Alanazi M.M., Shahid M., Alwetaid M.Y., Hussein M.H. (2023). Upregulation of Inflammatory Mediators in Peripheral Blood CD40^+^ Cells in Children with Autism Spectrum Disorder. Int. J. Mol. Sci..

[37] Zheng Y., Prince N.Z., Marzal L.N.P., Ahmed S., Garssen J., Pardo P.P., Kraneveld A.D. (2022). The Autism Spectrum Disorder-Associated Bacterial Metabolite *p*-Cresol Derails the Neuroimmune Response of Microglial Cells Partially via Reduction of ADAM17 and ADAM10. Int. J. Mol. Sci..

[38] Lee R.H., Mills E.A., Schwartz N., Bell M.R., Deeg K.E., Ruthazer E.S., Marsh-Armstrong N., Aizenman C.D. (2010). Neurodevelopmental effects of chronic exposure to elevated levels of pro-inflammatory cytokines in a developing visual system. Neural Dev..

[39] Tsai A.Y., Byndloss M.X., Seyffert N., Winter M.G., Young B.M., Tsolis R.M. (2022). Tumor Necrosis Factor Alpha Contributes to Inflammatory Pathology in the Placenta during Brucella abortus Infection. Infect. Immun..

[40] Romanowska-Próchnicka K., Felis-Giemza A., Olesińska M., Wojdasiewicz P., Paradowska-Gorycka A., Szukiewicz D. (2021). The Role of TNF-α and Anti-TNF-α Agents during Preconception, Pregnancy, and Breastfeeding. Int. J. Mol. Sci..

[41] Carpentier P.A., Dingman A.L., Palmer T.D. (2011). Placental TNF-α Signaling in Illness-Induced Complications of Pregnancy. Am. J. Pathol..

[42] Ashwood P. (2023). Preliminary Findings of Elevated Inflammatory Plasma Cytokines in Children with Autism Who Have Co-Morbid Gastrointestinal Symptoms. Biomedicines.

[43] Rose D.R., Yang H., Careaga M., Angkustsiri K., Van de Water J., Ashwood P. (2020). T cell populations in children with autism spectrum disorder and co-morbid gastrointestinal symptoms. Brain Behav. Immun. Health.

[44] Vargas D.L., Nascimbene C., Krishnan C., Zimmerman A.W., Pardo C.A. (2005). Neuroglial activation and neuroinflammation in the brain of patients with autism. Ann. Neurol..

[45] Li X., Chauhan A., Sheikh A.M., Patil S., Chauhan V., Li X.-M., Ji L., Brown T., Malik M. (2009). Elevated immune response in the brain of autistic patients. J. Neuroimmunol..

[46] Goines P.E., Ashwood P. (2013). Cytokine dysregulation in autism spectrum disorders (ASD): Possible role of the environment. Neurotoxicol. Teratol..

[47] Gomarasca M., Banfi G., Lombardi G. (2020). Myokines: The endocrine coupling of scheletal muscle and bone. Adv. Clin. Chem..

[48] Bickel M. (1993). The role of interleukin-8 in inflammation and mechanisms of regulation. J. Periodontol..

[49] Matsushima K., Yang D., Oppenheim J.J. (2022). Interleukin-8: An evolving chemokine. Cytokine.

[50] Bernhard S., Hug S., Stratmann A.E.P., Erber M., Vidoni L., Knapp C.L., Thomaß B.D., Fauler M., Nilsson B., Ekdahl K.N. (2021). Interleukin 8 Elicits Rapid Physiological Changes in Neutrophils That Are Altered by Inflammatory Conditions. J. Innate Immun..

[51] Zhang J., Bai C. (2017). Elevated Serum Interleukin-8 Level as a Preferable Biomarker for Identifying Uncontrolled Asthma and Glucocorticosteroid Responsiveness. Tanafos.

[52] Shen Y., Li Y., Shi L., Liu M., Wu R., Xia K., Zhang F., Ou J., Zhao J. (2021). Autism spectrum disorder and severe social impairment associated with elevated plasma interleukin-8. Pediatr. Res..

[53] Robinson-Agramonte M.d.L.A., García E.N., Guerra J.F., Hurtado Y.V., Antonucci N., Semprún-Hernández N., Schultz S., Siniscalco D. (2022). Immune Dysregulation in Autism Spectrum Disorder: What Do We Know about It?. Int. J. Mol. Sci..

[54] Yamauchi T., Makinodan M., Toritsuka M., Okumura K., Kayashima Y., Ishida R., Kishimoto N., Takahashi M., Komori T., Yamaguchi Y. (2021). Tumor necrosis factor-alpha expression aberration of M1/M2 macrophages in adult high-functioning autism spectrum disorder. Autism Res..

[55] Jian J., Li L.-G., Zhao P.-J., Zheng R.-J., Dong X.-W., Zhao Y.-H., Yin B.-Q., Li S., Cheng H., Li H.-L. (2023). Mouse nerve growth factor suppresses neuronal apoptosis in valproic acid-induced autism spectrum disorder rats by regulating the phosphoinositide-3-kinase/serine/threonine kinase signaling pathway. Pharmacogenet. Genom..

[56] Onore C., Careaga M., Ashwood P. (2012). The role of immune dysfunction in the pathophysiology of autism. Brain Behav. Immun..

[57] Jiang N.M., Cowan M., Moonah S.N., Petri W.A. (2018). The Impact of Systemic Inflammation on Neurodevelopment. Trends Mol. Med..

[58] Fujitani M., Miyajima H., Otani Y., Liu X. (2022). Maternal and Adult Interleukin-17A Exposure and Autism Spectrum Disorder. Front. Psychiatry.

[59] Xie J., Huang L., Li X., Li H., Zhou Y., Zhu H., Pan T., Kendrick K.M., Xu W. (2017). Immunological cytokine profiling identifies TNF-α as a key molecule dysregulated in autistic children. Oncotarget.

[60] Zhao H., Zhang H., Liu S., Luo W., Jiang Y., Gao J. (2021). Association of Peripheral Blood Levels of Cytokines with Autism Spectrum Disorder: A Meta-Analysis. Front. Psychiatry.

[61] Xu N., Li X., Zhong Y. (2015). Inflammatory Cytokines: Potential Biomarkers of Immunologic Dysfunction in Autism Spectrum Disorders. Mediat. Inflamm..

