# Supplementary material for: Immunological Biomarkers in Autism Spectrum Disorder: The Role of TNF-Alpha and Dependent Trends in Serum IL-6 and CXCL8
Source: Life (Basel). 2024 Sep 22;14(9):1201. doi: 10.3390/life14091201 (PMC11432970; doi:10.3390/life14091201)
Supplement: Supplementary file 1 [file life-14-01201-s001.zip › life-3156706-supplementary.pdf]

Table S1. Mean and standard deviation of serum interleukin 6 (IL-6), interleukin 8 (CXCL8), and tumor necrosis factor alpha (TNF-alpha) in the study group and the control group, ages between 2 and 12 years old.

Cytokines	Mean ± SD		p - value
	Study group 2 - 12 years	Control group 2 – 12 years	
TNF - alpha	7.980 ± 2.428	7.043 ± 2.418	0.100
CXCL8	9.064 ± 2.335	8.503 ± 2.600	0.343
IL - 6	1.665 ± 0.535	1.645 ± 0.406	0.592

CXCL8 = interleukin 8; Il-6 = interleukin 6; SD = standard deviation; P- value = significance at p < 0.05

Table S2. Mean and standard deviation of serum tumor necrosis factor alpha (TNF - alpha), interleukin 8 (CXCL8) and interleukin 6 (IL-6), concentrations by age in the study group and the control group.

Cytokines	Mean ± SD				p - value
	Study group		Control group		
	Age < 5 years	Age > 5 years	Age < 5 years	Age > 5 years	
TNF – alpha	9.165 ± 2.648	7.033 ± 1.775	9.437 ± 2.780	6.172 ± 1.589	< 0.001
CXCL8	8.877 ± 2.288	9.215 ± 2.862	8.467 ± 2.445	8.515 ± 2.353	0.780
IL – 6	1.713 ± 0.592	1.6506 ± 0.49	1.513 ± 0.038	1.696 ± 0.470	0.701

CXCL8 = interleukin 8; Il-6 = interleukin 6; SD = standard deviation; range age between 2 and 12 years old; P- value significance < 0.05.

Table S3. Mean and standard deviation of serum tumor necrosis factor alpha (TNF - alpha), interleukin 8 (CXCL8) and interleukin 6 (IL-6), concentrations by age in the study group and the control group.

Cytokines	Mean ± SD				p - value
	Study group		Control group		
	Age < 5 years	Age > 5 years	Age < 5 years	Age > 5 years	
TNF – alpha	9.165 ± 2.648	7.033 ± 1.775	9.437 ± 2.780	6.172 ± 1.589	< 0.001
CXCL8	8.877 ± 2.288	9.215 ± 2.862	8.467 ± 2.445	8.515 ± 2.353	0.780
IL – 6	1.713 ± 0.592	1.6506 ± 0.49	1.513 ± 0.038	1.696 ± 0.470	0.701

CXCL8 = interleukin 8; Il-6 = interleukin 6; SD = standard deviation; range age between 2 and 12 years old; P- value significance < 0.05.

Table S4. Statistical analysis of Interleukin 6, Interleukin 8 and Tumor Necrosis Factor alpha with mean, standard deviation, median and interquartile range (IQR) results..

Statistic	INTERLEUKIN 6	INTERLEUKIN 6 | control group	INTERLEUKIN 6 | study group
No. of observations	75	30	45
Mean	1,6654	1,6459	1,6780
Standard deviation (n-1)	0,4866	0,4067	0,5360
Minimum	1,5000	1,5000	1,5000
1st Quartile	1,5000	1,5000	1,5000
Median	1,5000	1,5000	1,5000
3rd Quartile	1,5000	1,5000	1,5000
Maximum	4,0400	3,1600	4,0400

Statistic	INTERLEUKIN 6	INTERLEUKIN 6 | control group	INTERLEUKIN 6 | study group
Median(IQR)	1.50 (1.50 - 1.50)	1.50 (1.50 - 1.50)	1.50 (1.50 - 1.50)

Statistic	INTERLEUKIN 8	INTERLEUKIN 8 | control group	INTERLEUKIN 8 | study group
No. of observations	75	30	45
Mean	8,8401	8,5030	9,0649
Standard deviation (n-1)	2,4968	2,3353	2,6002
Minimum	5,0000	5,0000	5,7300
1st Quartile	6,7700	6,4950	7,0400
Median	8,4100	8,7750	8,4100
3rd Quartile	10,1450	9,6925	10,6000

Statistic	INTERLEUKIN 8	INTERLEUKIN 8 | control group	INTERLEUKIN 8 | study group
Median(IQR)	8.41 (6.77 - 10.15)	8.78 (6.50 - 9.69)	8.41 (7.04 - 10.60)

Maximum	16,0000	14,2000	16,0000

Statistic	TNF alpha	TNF alpha | control group	TNF alpha | study group
No. of observations	75	30	45
Mean	7,6053	7,0433	7,9800
Standard deviation (n-1)	2,4521	2,4190	2,4284
Minimum	4,0000	4,0000	4,0000
1st Quartile	5,7000	5,5000	6,4000
Median	7,1000	6,4000	7,5000
3rd Quartile	9,0500	8,0500	10,1000
Maximum	14,6000	14,6000	14,1000

Statistic	TNF alpha	TNF alpha | control group	TNF alpha | study group
Median(IQR)	7.10 (5.70 - 9.05)	6.40 (5.50 - 8.05)	7.50 (6.40 - 10.10)
